# Structural and biochemical basis of ROC-dependent activation of LRRK2

**DOI:** 10.21203/rs.3.rs-8735353/v1

**Published:** 2026-02-02

**Authors:** Yangshin Park, Chunxiang Wu, Kayla Tennessen, Li Wan, Neo C. Hoang, Cardea W. Hoang, Jingling Liao, Quyen Q. Hoang

**Affiliations:** 1 Department of Biochemistry, Molecular Biology, and Pharmacology, Indiana University School of Medicine, Indianapolis, IN 46202,; 2 The Stark Neurosciences Research Institute, Indiana University School of Medicine, Indianapolis, IN 46202,; 3 Current address: Department of Molecular Biophysics and Biochemistry, Yale University, New Haven, CT, USA.; 4 Current address: School of Life and Health Sciences, Hainan Province Key Laboratory of One Health, Collaborative Innovation Center of Life and Health, Hainan University, Haikou, Hainan 570228, China.; 5 Current address: Academy of Nutrition and Health, School of Public Health, Wuhan University of Science and Technology, Wuhan, 430065, People’s Republic of China,; 6 Department of Neurology, Indiana University School of Medicine, Indianapolis, IN 46202.

**Keywords:** LRRK2, Parkinson’s disease, GTPase, Kinase, X-ray crystallography, Cryo-EM

## Abstract

Mutations in leucine-rich repeat kinase 2 (LRRK2) are the most common cause of familial Parkinson’s disease, yet the molecular mechanism governing LRRK2 activation remains incompletely understood. LRRK2 is a large multidomain enzyme whose kinase activity is regulated by intramolecular interactions and by its Ras of complex proteins (ROC) GTPase domain. Here, we combine cryo–electron microscopy, X-ray crystallography, and structure-guided biochemical perturbations to define how ROC conformational switching regulates LRRK2 activation. Cryo-EM reconstructions reveal that monomeric full-length LRRK2 samples three distinct conformational states—autoinhibited, intermediate, and activated— indicating that large-scale activation-associated rearrangements can occur through an intrinsic intramolecular pathway, independently of Rab29 binding, higher-order oligomerization, or membrane association. A 1.6 Å crystal structure of an extended ROC construct reveals intrinsic conformational plasticity within the GTPase switch regions that likely underlies these transitions. Structure-guided disulfide engineering identifies a functional coupling between residue R1441 and Switch II that directly modulates GTPase activity in both isolated ROC and full-length LRRK2. Disruption of this coupling phenocopies the disease-associated R1441H mutation. Together, these findings establish ROC as a dynamic conformational engine that drives a multistep intramolecular activation mechanism in LRRK2, providing mechanistic insight into how pathogenic mutations promote aberrant kinase activation.

## Introduction

Mutations in leucine-rich repeat kinase 2 (LRRK2) are the most common genetic cause of Parkinson’s disease (PD), with pathogenic variants consistently associated with elevated kinase activity and neurodegeneration (1–3). LRRK2 is a large, multidomain enzyme composed of N-terminal solenoid repeat domains—armadillo (ARM), ankyrin (ANK), and leucine-rich repeats (LRR)—followed by a C-terminal catalytic core comprising a Ras of complex proteins (ROC) GTPase domain, a C-terminal of ROC (COR) domain, a protein kinase domain, and a WD40 β-propeller (4–6). Despite extensive genetic, biochemical, and structural studies, the molecular mechanism by which LRRK2 transitions from an inactive to an active state remains incompletely understood (7).

Recent cryo–electron microscopy (cryo-EM) structures have provided important insights into the overall architecture of LRRK2 and revealed extensive intramolecular interactions that constrain the kinase domain in an autoinhibited conformation. In these structures, the LRR domain wraps around the kinase domain, occluding access to the active site, while the COR and WD40 domains engage the kinase lobes and stabilize an open, inactive configuration (5). Other studies have suggested that activation involves the disengagement of inhibitory N-terminal domains and rearrangements within the ROC–COR–kinase–WD40 module, potentially driven by Rab29 binding, oligomerization, and membrane association (8). However, how these events are coordinated at the molecular level and how nucleotide-dependent regulation by the ROC GTPase domain is integrated into this process remains unclear (6, 7).

A substantial body of evidence implicates the ROC domain as a key regulator of LRRK2 activity. GTP binding and hydrolysis are required for normal kinase function (9–11). Moreover, several PD-associated mutations cluster within ROC, most notably at residue R1441, which consistently increase kinase activity in cellular and animal models (12–15). Notably, R1441 is located distal from the nucleotide-binding pocket, leaving unresolved how mutations at this site impair GTPase activity and promote pathological kinase activation.

Previous work from our group and others demonstrated that the isolated ROC domain is a *bona fide* GTPase that undergoes nucleotide-dependent conformational switching, including monomer–dimer transitions, and that the PD-associated mutations at R1441 impair GTP hydrolysis and stabilize the “on” conformation (16–18). These observations led to the hypothesis that ROC functions as a conformational switch that regulates LRRK2 activation through long-range intramolecular signaling (6). However, whether these properties of ROC are preserved in the context of full-length LRRK2, and how conformational changes in ROC propagate through the multidomain architecture to control kinase activation, remain open questions.

Here, we address these questions through an integrated structural and biochemical analysis of ROC and full-length LRRK2. Using cryo-EM, we show that monomeric full-length LRRK2 samples at least three distinct conformational states—autoinhibited, intermediate, and activated—demonstrating that large-scale activation-associated rearrangements can occur independently of Rab29 binding, higher-order oligomerization, or membrane binding. We further determined a 1.6 Å crystal structure of an extended ROC construct, revealing intrinsic conformational plasticity within the switch regions that likely underlies its regulatory function. Finally, through targeted disulfide engineering and biochemical assays, we identify a functional coupling between residue R1441 and Switch II that modulates GTPase activity in both isolated ROC and full-length LRRK2, and show that disruption of this coupling phenocopies the disease-associated R1441H mutation.

Together, these findings support ROC as a central conformational regulator that drives a multistep intramolecular activation mechanism, providing a structural and mechanistic framework linking nucleotide dependent ROC switching to kinase activation and the molecular basis of pathogenic mutation.

## Results and Discussion

### Nucleotide-dependent conformational changes and GTPase activity of ROC in the context of full-length LRRK2

**Figure 1.**
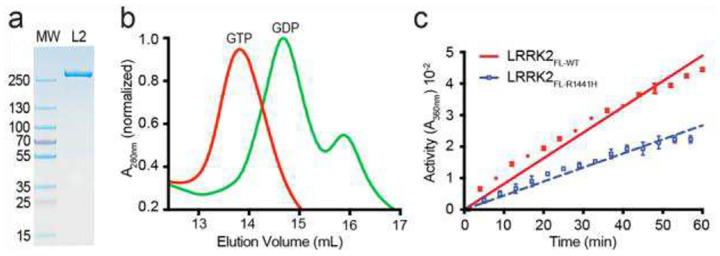
Purification and biochemical activity of full-length LRRK2. **a)** SDS-PAGE analysis demonstrating the purity of the purified LRRK2 sample (L2). **b)** Size-exclusion chromatography elution profile of LRRK2 on a Superose 6 column, illustrating nucleotide-dependent conformational changes. **c)** GTPase activity assay showing that the PD-associated R1441H variant (blue, dashed) exhibits reduced GTP hydrolysis compared to the wild-type (red).

Previously, we showed that a construct of the ROC domain spanning residues 1329 to 1520 (we called ROC_ext_) is a *bona fide* G-protein that has GTPase activity and undergoes nucleotide-dependent conformational switching – both of which are impaired by the Parkinson’s disease-associated mutations at R1441 and N1437 (16–18). To test whether these properties occur in the context of full-length LRRK2, we expressed full-length LRRK2 in insect cells and purified it to homogeneity, using an immobilized anti-Flag antibody column followed by size-exclusion chromatography (Fig. 1) for biochemical and structural analyses.

GTP-bound LRRK2 eluted from the Superose 6 size-exclusion column with a smaller volume than the GDP-bound sample (13.7 ml and 14.6 ml, respectively) (Fig. 1b), indicating that the GDP-bound LRRK2 adopts a more compact conformation than the GTP-bound form, reminiscent of the nucleotide-dependent conformational changes observed with ROC_ext_ (16, 17).

Using a GTPase assay, we observed that full-length LRRK2 carrying the PD-associated substitution R1441H exhibits lower activity than that of the wild-type (WT) (Fig. 1c), consistent with our previous reports for ROC_ext_ (16–18).

Taken together, these results recapitulate the biochemical properties of ROC_ext_ in the context of full-length LRRK2, indicating that the activity of the isolated ROC_ext_ construct represents its activity in the full-length context and suggesting that ROC conformational changes modulate the overall structure of full-length LRRK2.

### Cryo-EM reveals three structurally distinct conformations of full-length LRRK2

In earlier reports, we showed that under low nucleotide conditions, ROC_ext_ samples both GTP-bound and GDP-bound conformations (16, 17). To investigate how a low-nucleotide condition may affect the conformation of full-length LRRK2, we used cryo-EM to assess the conformational heterogeneity of full-length LRRK2. Purified LRRK2 was applied to glow-discharged gold foil grids (Quantifoil UltrAuFoil R1.2/1.3) and vitrified in liquid ethane using a Vitrobot Mark IV. We collected 2,945 movies on a Glacios microscope operating at 200 kV, with a physical pixel size of 0.93 Å, a defocus range of −0.1 to −3.8 μm, and a total exposure of 50 e^−^/Å^2^. Image processing was performed in CryoSPARC v4.7.1 (19). Iterative particle picking with Topaz (20), followed by 2D classification, *ab initio* reconstruction, resolved three distinct 3D classes (Fig. S1). Non-uniform refinement produced maps with average resolutions (GSFSC) of 7.8 Å, 7.1 Å, and 8.9 Å (Fig. S2).

**Figure 2.**
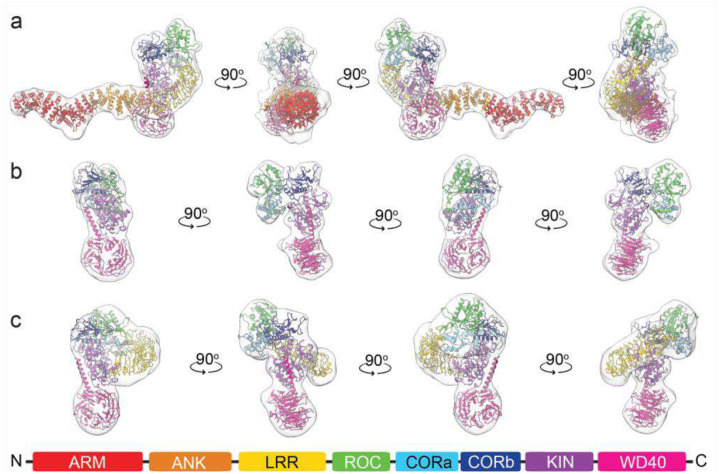
Cryo-EM structures of monomeric full-length LRRK2. **a)** A 7.8Å map (light gray, semitransparent) of monomeric full-length LRRK2 showing all domains with Arm (red), Ank (orange), LRR (yellow), ROC (green), CORa (light blue), CORb (blue), Kinase (purple), and WD40 (magenta) in ribbon presentation. **b)** A 7.1 Å map showing the ROC-COR-Kinase-WD40 segment of LRRK2. **c)** An 8.9 Å map showing the LRR-ROC-COR-Kinase-WD40 segment of LRRK2.

Despite the modest resolutions (7.1–8.9 Å), all three volumes were clearly identifiable as LRRK2, and models could be fitted into the map unambiguously. Volume 1 exhibited the characteristic “e”-shaped architecture of the autoinhibited full-length protein. Chain E from PDB ID 8OF9 was manually docked into this map and refined as a rigid body in Phenix (Fig. 2a) (21, 22). Visual inspection confirmed an excellent fit (CC½_mask for each domain ranged from 0.50 to 0.73).

The same initial model was used for volume 2, which has clear density for the ROC–COR–Kinase–WD40 segment but lacks the N-terminal Arm–Ank–LRR domains. In contrast to volume 1, rigid-body refinement of the Roc-COR-Kinase-WD40 segment suggested that the orientations of the domains relative to one another required adjustment. Each domain was separated, assigned a unique chain ID, and refined independently as a rigid body in Phenix (22). All domains fit the density well except the kinase N-lobe, which required an additional round of rigid-body refinement after being severed from the C-lobe and allowed to move independently. This yielded an excellent fit to the EM map (CC½_mask ranged from 0.65 to 0.70) (Fig. 2b).

We applied the same model-fitting strategy for volume 3, which spans the LRR–ROC–COR–Kinase–WD40 region. As with volume 2, domain rearrangements were necessary to achieve an optimal fit (CC½_mask ranged from 0.54 to 0.71) (Fig. 2c).

**Figure 3.**
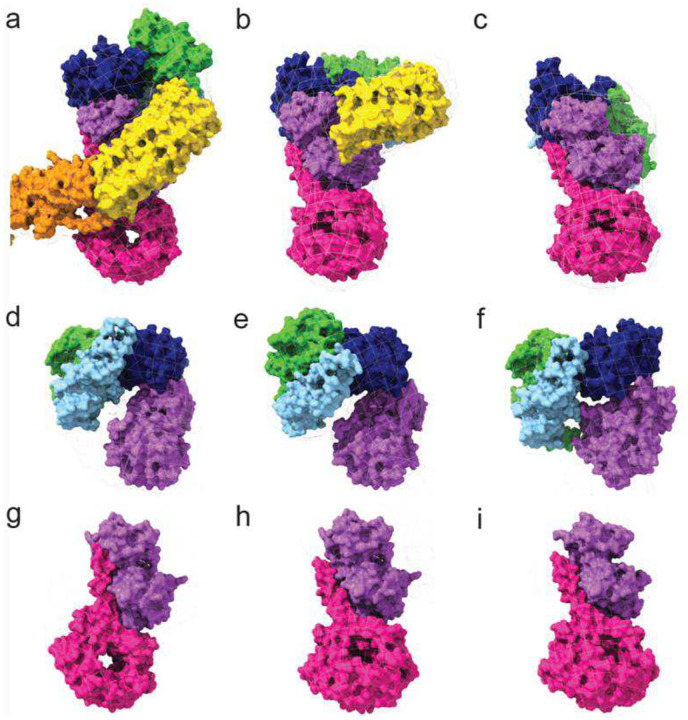
Arrangement of domains in monomeric full-length LRRK2. Surface presentation of the autoinhibited (**a**, **d**, **g**), intermediate (**b**, **e**, **h**), and activated (**c**, **f**, **i)** models of LRRK2 with the EM map mesh. **a**), **b**), and **c**) show disengagement of the Ank domain (orange) from the WD40 (magenta), followed by disengagement of the LRR domain (yellow) from the kinase domain (purple), opening access to its active site. **d**), **e**), and **f**) show the arrangement of ROC-COR domains with respect to the Kinase domain, illustrating the ROC-CORa-CORb (green-light blue-blue) module rotating about the kinase domain (purple) and clamping it in an active conformation (**f**). **g**), **h**), and **i**) illustrate that the C-terminal helix of WD40 (magenta) is disengaged from the N-lobe of the kinase domain (purple) in its active conformation.

Comparison of the three models revealed several mechanistically informative differences. Model 1 is composed of the entire full-length LRRK2, including the N-terminal Arm–Ank–LRR segment, whereas model 3 lacks the Arm-Ank domains, and model 2 lacks Arm-Ank-LRR (Fig. 3a–c). Additionally, the LRR domain in model 3 is shifted away from the kinase domain and partially undocked from it compared to model 1 (Fig. S3).

Taken together with prior studies, these observations lead us to propose that model 3 represents an intermediate state between the fully docked conformation (model 1) and the fully undocked state (model 2). These data support a stepwise undocking mechanism in which the LRR domain first disengages from the WD40 domain and subsequently separates from the kinase domain.

Significant structural rearrangements are also observed downstream of the LRR domain. In model 2, the ROC–COR assembly rotates about the Kinase domain by ~40 degrees relative to its position in model 1 and clamps the N- and C-lobe of the kinase together, consistent with adoption of a closed, catalytically competent configuration (Fig. 3d–f and Fig. S4). In contrast, the ROC–COR domains in the intermediate model exhibit a partial displacement, and the kinase domain remains in the open, inactive conformation observed in model 1. Although the intermediate conformation is defined here as a structurally distinct class, we cannot exclude the possibility that it represents a metastable state within a broader conformational continuum sampled by LRRK2 under low-nucleotide conditions.

Structural changes within the kinase–WD40 region are subtle but functionally significant. In the autoinhibited state, the C-terminal helix of the WD40 domain is wedged against the backside of the kinase domain (opposite the active site), effectively locking the kinase, stabilizing an open, inactive conformation (Fig. 3g). In the activated state, this C-terminal helix disengages from the kinase N-lobe, allowing the N-lobe to rotate toward the C-lobe and adopt a closed, catalytically competent conformation (Fig. 3i).

These structures suggest a distinct activation mechanism. The prevailing model in the literature proposes that Rab29-dependent tetramerization activates two protomers, and the remaining two become activated upon membrane binding (8). In contrast, our data show that both the autoinhibited and activated conformations can occur as monomers and in the absence of Rab29 or membranes.

Furthermore, the intermediate conformation we identified supports a stepwise activation mechanism in which the N-terminal arm first disengages from the WD40 domain, followed by the LRR domain detaching from the kinase domain, and the ROC-COR module clamps the kinase domain in an active configuration. Given that we observed a full spectrum of LRRK2 conformations - from autoinhibited to intermediate to activated - in the absence of Rab29 or oligomerization, and our earlier work showing that isolated ROC domain (ROC_ext_) dynamically samples “on” and “off” conformations under low-nucleotide conditions, we propose that activation of LRRK2 is driven primarily by ROC-mediated intramolecular domain interactions and structural rearrangements. Importantly, the intrinsic intramolecular activation pathway described here does not preclude additional layers of regulation in cellular contexts. Rab29 binding, membrane association, and higher-order oligomerization may unction to bias the conformational ensemble of LRRK2 toward specific states or stabilize activated conformations under physiological conditions. In this view, ROC-mediated intramolecular rearrangements define a core activation mechanism that can be further modulated by extrinsic factors, rather than representing an alternative or mutually exclusive pathway.

While the resolutions of the cryo-EM reconstructions (7–9 Å) do not permit atomic-level interpretation, they are sufficient to define the relative positions and orientations of major domains within full-length LRRK2. Accordingly, the conformational states described here are based on rigid-body fitting of previously determined domain structures and are interpreted at the level of large-scale domain rearrangements rather than local structural changes. The observed transitions between autoinhibited, intermediate, and activated states therefore reflect differences in overall domain organization that are consistent with, but do not by themselves establish, the molecular details of activation. Within these constraints, the cryo-EM data support a model in which LRRK2 samples a discrete set of intramolecular conformations that differ in the degree of domain docking and kinase closure.

### Crystal structure of the ROC_ext_

Parkinson’s disease-associated mutations R1441H/G/C in ROC impair its GTPase activity and nucleotide-dependent conformational changes (Fig. 1 and (16, 17)). However, R1441 is not proximal to the nucleotide-binding pocket or catalytic center of ROC, leaving the structural basis for these defects unclear. To address this question, we undertook a detailed structural and functional characterization of ROC_ext_. We determined the crystal structure of ROC_ext_ to 1.6 Å resolution by X-ray crystallography. Initial phases were obtained by single-wavelength anomalous dispersion from selenomethionine-substituted crystals (SeMet-SAD), which diffracted to 3.7 Å, using SHARP(23), and were combined with phases calculated by molecular replacement using Phenix(21) from a 1.9 Å native dataset. To improve crystal quality and achieve higher resolution, we employed surface engineering by substituting two solvent-exposed lysine residues (K1460A and K1463A). The surface-engineered mutant (ROC_ext_KA_) retained wild-type activity and structure (Fig. S5). Data from crystals of the surface-engineered protein were collected to 1.6 Å resolution and used for model building and refinement. A continuous polypeptide chain encompassing residues 1331–1518 - nearly the entire ROC_ext_ construct (1329–1520) - was unambiguously modeled into the electron density map and refined to convergence with R-factor and R-free values of 14% and 16%, respectively (Sup. Table 1).

The asymmetric unit contains a homodimer of ROC_ext_ related by twofold rotational symmetry. The interactions at the dimer interface are extensive, burying 6461 Å^2^ of surface area (Fig. S6a–c). The intermolecular interactions are mediated chiefly by the regions known as Switch I, Switch II, and the InterSwitch, herein collectively referred to as the Switch regions (Fig. S6b,c). Notably, the dimer adopts an unusual conformation in which the InterSwitch is flipped open and inserted into the opposing protomer in trans, resulting in the two molecules wrapping around each other in a pretzel-like arrangement (Fig. S6a).

**Figure 4.**
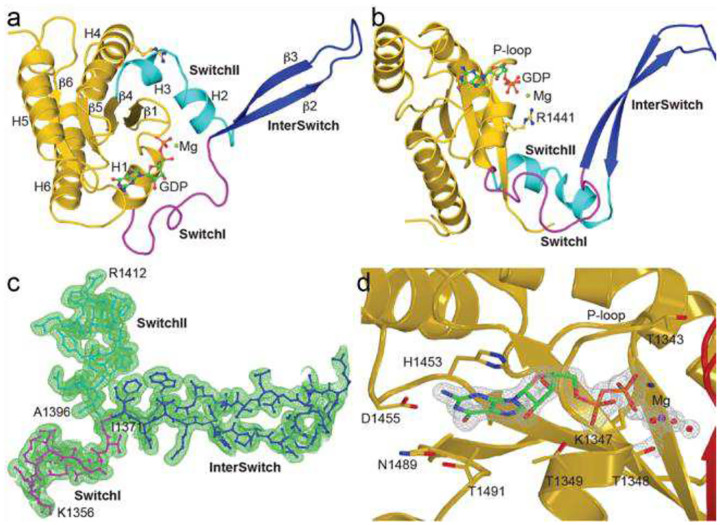
Structure of ROCext. **a**) and **b**) Crystal structure of ROC dimer (1.6 Å) shown in ribbon presentations (orange), highlighting GTPase Switch I (magenta), InterSwitch (blue), and Switch II (teal). GDP is shown as a rod-and-ball model. **c**) and **d**) electron-density map (2FoFc) of the Switch regions and GDP, respectively, contoured at 1.0 σ

A similar dimeric conformation has been previously observed in *M. Musculus* Rab27b, which showed a similar extended InterSwitch structure that occurs in the absence of its effector Slac2-a, and adopts a typical G-protein conformation upon binding its effector (24, 25). By analogy, we propose that the extended InterSwitch observed in ROC_ext_ arises from the absence of its physiological binding partner, the COR domain. Consistent with this interpretation, all available structures of full-length LRRK2 or ROC–COR–KIN–WD40 assemblies show ROC adopting a canonical small GTPase fold (5, 26). Aside from the extended InterSwitch, the overall tertiary structure of ROC_ext_ closely resembles that of classical small G-proteins with clearly defined Switch I, Switch II, P-loop, and G-binding motifs (Fig. 4a–d), as well as a bound GDP molecule (Fig. 4d).

### The crystal dimer exists in solution

Previously, we showed that ROC_ext_ undergoes nucleotide-dependent conformational changes, specifically dimerization upon GDP binding and monomerization upon GTP binding (17), suggesting that the dimeric interface may involve the two nucleotide-binding switches. However, the crystal structure of ROC_ext_ reveals that the dimer is predominantly stabilized by five hydrogen bonds formed between two β-strands at the interface – a β-strand (β3) in the InterSwitch of one protomer and another β-strand (β1) in the core region of the opposing protomer (Fig. S7b), which are not directly involved in nucleotide binding.

**Figure 5.**
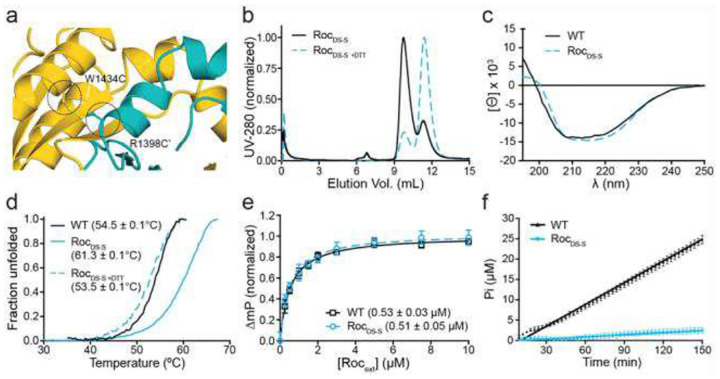
Structure and function of ROCext-DSS. **a**) Ribbon presentation of ROCext-DSS showing the engineered disulfide bond as a ball-and-stick presentation (yellow). **b**) Size-exclusion chromatography showing ROCext-DSS dimers (solid black) dissociating into monomers upon adding DTT (dashed blue). **c**) CD spectroscopy showing ROCext-DSS (dashed blue) exhibits essentially identical secondary structure to WT (black solid). **d**) Thermal denaturation assay showing ROCext-DSS (solid blue) is more thermostable than WT (solid black) and reverts to WT melting temperature upon adding DTT (dashed blue). **e**) Fluorescence polarization assay showing ROCext-DSS (dashed blue) exhibits essentially identical affinity for GDP as WT (solid black). **f**) GTPase assay showing ROCext-DSS (blue) has no detectable activity.

To test whether the ROC_ext_ crystal dimer also exists in solution, we substituted residue R1398 and W1434, which are positioned adjacent to each other across the dimer interface, with cysteine residues to enable formation of an engineered disulfide bond (Fig. 5a, Fig. S7). Size-exclusion chromatography revealed that the resulting disulfide-stabilized ROC_ext_ construct (ROC_ext-DSS_) adopts a stable dimeric conformation reminiscent of the GDP-bound wild-type (WT) ROC_ext_ we previously reported (16, 17). Under reducing conditions, which disrupt disulfide bonds, the ROC_ext-DSS_ dimer dissociated into monomers (Fig. 5b). Circular dichroism spectroscopy (CD) revealed no significant differences in secondary structure between the DSS mutant and the WT dimers (Fig. 5c), indicating that the disulfide bond stabilizes the dimer without introducing detectable structural perturbations.

We measured the thermal stability of ROC_ext_ in the presence and absence of DTT and observed that the DSS mutant (Tm = 61.3 °C) is 6.8 °C more stable than WT (Tm = 54.5 °C); however, upon addition of DTT, the stability of ROC_ext-DSS_ (Tm = 53.3 °C) became comparable to that of WT (Fig. 5d), indicating that the disulfide bond stabilizes the dimeric conformation, and, together, suggest that the crystal dimer is representative of the solution-state dimer.

To test whether the GDP-binding site is preserved in the DSS mutant, we determined its affinity for GDP using fluorescence polarization (16). We observed that ROC_ext-DSS_ exhibited essentially identical affinity for GDP compared with wild-type ROC_ext_ (0.51 ± 0.05 μM and 0.53 ± 0.03 μM, respectively) (Fig. 5e), indicating that nucleotide binding is not perturbed by the engineered disulfide bond. However, in contrast to wild-type ROC_ext_, ROC_ext-DSS_ exhibited no detectable GTPase activity under oxidizing conditions (Fig. 5f), consistent with our previous report that monomerization of ROC_ext_ is essential for GTP hydrolysis (16, 17).

Taken together, these results support the conclusion that the *in crystallo* dimer is structurally equivalent to the dimer in solution and provide a structural framework for understanding the coupling between nucleotide-dependent conformational changes and GTPase activity we previously reported for ROC_ext_ (16–18). Although the ROC_ext_ dimer observed *in crystallo* lacks the COR domain and therefore does not recapitulate the full intramolecular context of LRRK2, the extensive interface mediated by the Switch regions provides a structural framework for understanding how nucleotide-dependent conformational changes within ROC can influence long-range signaling. In full-length LRRK2, analogous switch-mediated interactions are likely repurposed for intramolecular communication rather than stable dimer formation, consistent with the dynamic rearrangements observed by cryo-EM.

### Structural basis for residue R1441 activity

Previously, we reported that Parkinson’s disease-associated mutations at residue R1441 impair both dimer formation and GTPase activity (16, 17). To gain insights into the mechanism of these impairments, we examined the crystal structure and found that R1441 extends across the dimer interface to engage the opposing protomer (Fig. S8). This interaction is centered on an arginine–π stacking interaction between R1441 and F1401 that orients the two ω-amines for hydrogen bonding with the backbone carbonyl oxygen of F1401 and the side chain hydroxyl of T1404 (Fig. S8). The side chain of R1441 fits into a pocket formed by F1401 and T1404 with high geometric and van der Waals complementarity (Fig. S8).

Given the strength and complementarity of the interactions, it is unsurprising that the Parkinson’s disease-associated mutations R1441G/C/H disrupt the dimer (16, 17). Notably, even substitution with lysine—a relatively conservative replacement that preserves a positively charged amine and a long aliphatic side chain—results exclusively in a monomeric conformation (Fig. S8c). Despite being monomeric, the R1441K mutant also displays markedly reduced GTPase activity, comparable to that observed for the PD-associated mutations (Fig. S8d). These observations indicate that residue R1441 is essential not only for conformational dynamics but also directly for ROC_ext_ GTPase activity.

To further investigate the role of R1441 in the catalytically active, GTP-bound conformation, we constructed a theoretical model of GTP-bound ROC (ROC_GTP_MD_). Because extensive crystallization attempts of ROC with GTP were unsuccessful, this model was generated by combining the protein core from our crystal structure with Switch I and II regions grafted from a homology model based on the GTP-bound Ras structure (PDB ID: 6Q21), followed by energy minimization and molecular dynamics simulations using NAMD (27, 28). In the ROC_GTP_MD_ model, the side chain of R1441 engages Switch II near residue M1409, suggesting a role in modulating Switch II conformation and GTPase activity.

**Figure 6.**
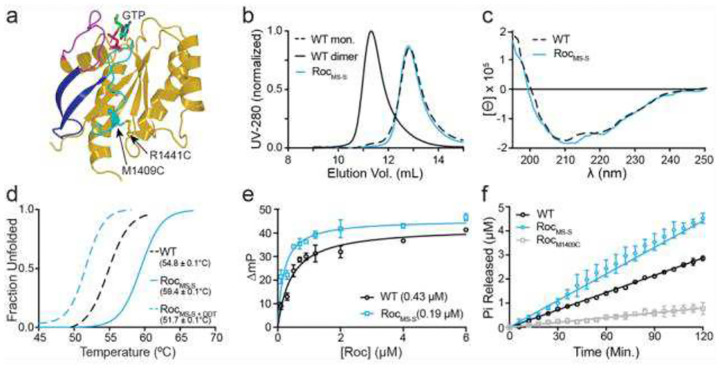
Structure and function of ROC_ext-MSS_. **a**) Ribbon presentation of a theoretical model of GTP-bound ROC monomer (ROC_ext-MSS_) showing the engineered disulfide bond as a rod-bond presentation (yellow). **b**) Size-exclusion chromatography showing ROC_ext-MSS_ monomer (blue) relative to WT dimer (black solid) and monomer (dashed black). **c**) CD spectroscopy showing ROC_ext-MSS_ (blue) exhibits essentially identical secondary structure to WT (dashed black). **d**) Thermal denaturation assay showing ROC_ext-MSS_ (solid blue) is more thermostable than WT (dashed black) and became less stable upon adding DTT (dashed blue). **e**) Fluorescence polarization assay showing ROC_ext-MSS_ (blue) exhibits essentially similar affinity for GDP as WT (solid black). **f**) GTPase assay showing ROC_ext-MSS_ (blue) exhibits higher activity than WT (black), while the single M1409C substitution abolishes activity.

Guided by this model, we engineered a covalent linkage between R1441 and the Switch II by substituting residues M1409 and R1442 with cysteine (ROC_ext-MSS_) (Fig. 6a). Using the same characterization approaches applied to the disulfide-stabilized dimer ROC_ext_DSS_, we found that ROC_ext-MSS_ is entirely monomeric (Fig. 6b), retains wild-type secondary structure (Fig. 6c), exhibits increased thermal stability under oxidizing conditions (Fig. 6d), displays higher affinity for GDP (Fig. 6e), and, notably, shows increased GTPase activity relative to WT (Fig. 6f). Together, these results indicate that the interactions between residue R1441 and Switch II play a critical role in modulating nucleotide affinity and GTPase activity.

### R1441-Switch II interaction modulates GTPase activity in full-length LRRK2

**Figure 7.**
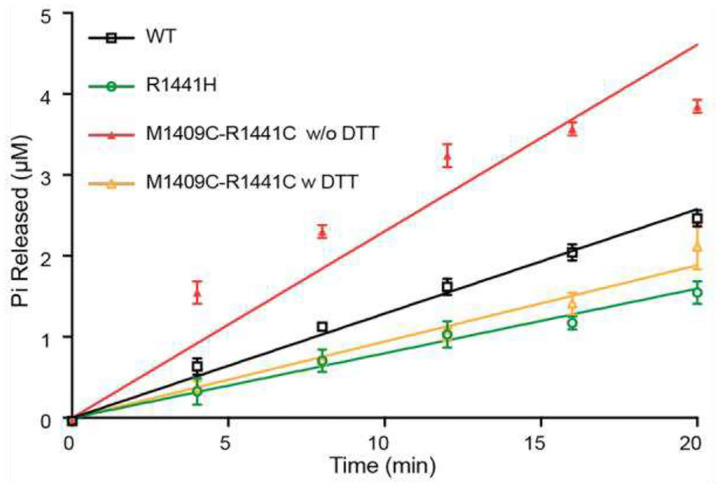
GTPase activity of full-length LRRK2. The R1141C-M1409C (red) mutant displays markedly higher activity than WT (black). However, in the presence of DTT, R1441C-M1409C shows similar activity to the PD-associated mutant R1441H.

To determine whether the R1441–Switch II coupling identified in ROC_ext_ is preserved in the context of full-length LRRK2, we introduced the corresponding cysteine substitutions (R1441C and M1409C) into full-length LRRK2 and measured GTPase activity in the presence or absence of DTT to control disulfide bond formation. In the absence of DTT, conditions that permit formation of the R1441C–M1409C disulfide bond, full-length LRRK2 exhibited a pronounced increase in GTPase activity relative to WT protein (Fig. 7). Disruption of the covalent linkage by addition of DTT abolished this enhancement and reduced GTPase activity to levels comparable to those observed for the Parkinson’s disease-associated R1441H mutant. Notably, this represents the first direct biochemical characterization of the R1441H mutation in full-length LRRK2 and reveals a marked reduction in GTPase activity relative to WT. Together, these results indicate that coupling between R1441 and Switch II is required for efficient GTP hydrolysis in full-length LRRK2 and that disruption of this interaction phenocopies the Parkinson’s disease-associated R1441H mutation, providing mechanistic insight into how pathogenic substitutions at R1441 impair LRRK2 GTPase activity.

### Conclusion

In this study, we provide structural and biochemical evidence that the ROC GTPase domain functions as a central conformational regulator of LRRK2 activation. By integrating cryo-EM analyses of full-length LRRK2, a high-resolution crystal structure of ROC_ext_, and targeted biochemical perturbations, we show that nucleotide-dependent conformational switching within ROC drives a multistep intramolecular activation process that culminates in kinase activation.

Cryo-EM reconstructions reveal that monomeric LRRK2 samples at least three distinct conformational states—autoinhibited, intermediate, and activated—demonstrating that large-scale activation-associated rearrangements can occur independently of Rab29 binding or higher-order oligomerization. The presence of an intermediate conformation supports a stepwise activation mechanism in which ROC–COR rearrangements precede and promote disengagement of inhibitory N-terminal domains and subsequent closure of the kinase active site.

The 1.6 Å crystal structure of ROC_ext_ provides a structural basis for this conformational plasticity, revealing switch regions that mediate both dimerization and intramolecular interactions. Within this framework, residue R1441 emerges as a critical regulatory node. Structural analysis shows that enforced coupling between R1441 and Switch II enhances GTPase activity in both isolated ROC_ext_ and full-length LRRK2, whereas disruption of this coupling reduces activity to levels comparable to those observed for the Parkinson’s disease–associated R1441H mutation.

**Figure 8.**
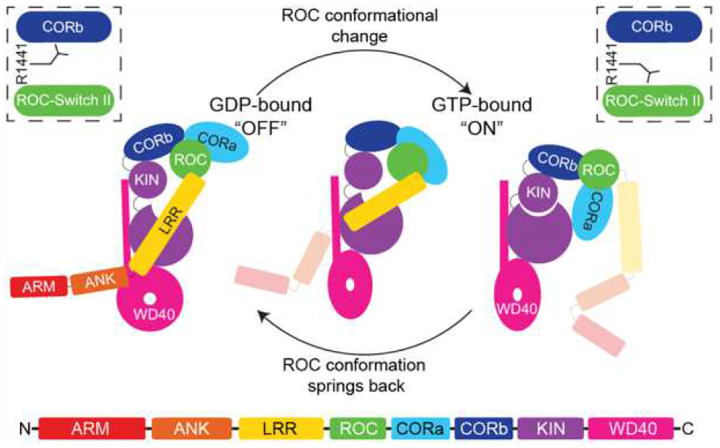
A model of ROC-mediated conformational change and activation of LRRK2. The apo form of LRRK2 samples at least 3 different conformations, ranging from the autoinhibited to the kinase-active state, as well as an intermediate conformation. The binding of GDP stabilizes the ‘off’ conformation of ROC and the autoinhibited conformation of LRRK2. GTP binding stabilizes the ‘on’ conformation of ROC and drives structural changes that lead to the kinase-active conformation of LRRK2.

Together, these findings support a model in which nucleotide-dependent conformational switching of ROC regulates LRRK2 activation by modulating long-range intramolecular interactions that control kinase domain accessibility and activity (Fig. 8). Pathogenic mutations at R1441 impair this regulatory coupling, thereby perturbing GTPase activity and contributing to aberrant kinase activation in Parkinson’s disease. By establishing ROC as a dynamic conformational engine rather than a passive regulatory appendage, this work provides a mechanistic framework for understanding LRRK2 regulation and insight into the mechanism of the defining ROC-COR module of the Roco family of proteins.

## Methods

### Protein Expression and Purification

An extended GTPase domain of LRRK2 consisting of residues 1329–1520 (ROC_ext_) was subcloned into a pETDuet-1 vector (Novagen, Merch KgaA, Darmstadt, Germany) using PCR cloning techniques. The resulting protein consisting of an N-terminal hexahistidine tag was expressed from Rosetta2 (DE3) *E. coli* (Novagen) by inducing with 0.5 mM isopropyl-β-D-thiogalactopyranoside (IPTG) for 16 hrs at 20 °C. Cells were harvested by centrifugation and lysed by sonication in a buffer containing 30 mM HEPES (pH 7.4), 250 mM NaCl, 10 mM MgCl_2_, 10 mM Glycine, 20 mM imidazole, 10 μM GDP, and 10% (v/v) Glycerol. Cell debris was cleared by ultra-centrifugation at 14,000 g (35,000 rpm, Beckman 45 Ti rotor). The supernatant was incubated with Ni-NTA Agarose (Invitrogen) for 2 hrs at 4 °C, then washed with lysis buffer (detailed above) and eluted with buffer containing 30 mM HEPES (pH 7.4), 250 mM NaCl, 10 mM MgCl_2_, 10 mM Glycine, 300 mM imidazole, 1 mM DTT, 10 μM GDP, and 10% Glycerol. The purified protein was then ‘polished’ by passing through a size-exclusion column (Superdex 200, GE Healthcare) in buffer containing 30 mM HEPES pH 7.4, 150 mM NaCl, 10 mM MgCl_2_, 10 mM Glycine, 1 mM DTT, and 10% Glycerol. The purified protein was then concentrated to ~15 mg/mL, flash frozen in liquid nitrogen and stored at −80 °C. The disulfide bond-stabilized ROC dimer (S-S), consisting of R1398C and W1434C double mutation, was subcloned in the pETDuet-1 vector and expressed from SHuffle T7 Express *lysY E. coli* (New England Biolabs Inc. MA, USA) by inducing with 0.5mM IPTG for 16 hrs at 20°C. The purification of the ROC S-S was performed as described for the wild-type.

The full-length human LRRK2 construct, consisting of a Flag tag at its N-terminus, was cloned into a pFL vector for expression in insect cell cultures. Recombinant LRRK2 Bacmid DNA was generated by transformation of competent DH10Bac E. coli cells using the MultiBac system (29). Recombinant baculovirus was isolated from Sf9 cell culture supernatant five days after transfection with LRRK2 Bacmid DNA. For recombinant protein expression, Hi5 cells was infected with various combinations of cloned viruses at a multiplicity of infection of 2–10. After 72 hr, the cells were harvested by centrifugation and stored as a pellet at −80°C until use. Cells were lysed by sonication in a buffer containing 50 mM Tris (pH 7.4), 300 mM NaCl, 10 mM MgCl2, 10 mM Glycine, 10 μM GDP, 0.006% LMNG, 10% (v/v) Glycerol and protease inhibitor cocktail (Thermo Scientific, USA). Cell debris was cleared by ultra-centrifugation at 14,000 g (35,000 rpm, Beckman 45 Ti rotor). The supernatant was incubated with anti-Flag M2 agarose (Sigma, USA) for 3 hrs at 4 °C, then washed with lysis buffer (detailed above) and eluted with buffer containing 50 mM Tris (pH 7.4), 300 mM NaCl, 10 mM MgCl2, 10 mM Glycine, 0.0002% LMNG, 10% (v/v) Glycerol and 3X FLAG Peptide(Fisher scientific, Apexbio technology LLC, USA). (To prepare a stock solution at a concentration of 5mg/ml, a working concentration of 100ug/ml.) The purified protein further purified on a size-exclusion chromatography (Superose 6 increase 10/300 GL, GE Healthcare) in 50 mM Tris (pH 7.4), 300 mM NaCl, 10 mM MgCl2, 10 mM Glycine, 0.0002% LMNG, 1mM DTT and 10% (v/v) Glycerol.

To determine the effect of GDP/GTP cycles in the full-length LRRK2, the purified protein (~2mg/ml) was incubated at room temperature with 20mM GDP or GTP for 4 hours. This incubation was done without the addition of EDTA. The incubated samples were then determined by size-exclusion chromatography (Superose 6 increase 10/300 GL, GE Healthcare).

### Expression and Purification of Selenomethionine Substituted Protein

SeMet ROC_ext_ was expressed from Rosetta2 (DE3) *E. coli* (Novagen). Rosetta2 (DE3) cells were grown in the M9 minimal medium. Methionine synthesis was inhibited by adding 100 mg of Lys, Phe, Thr (Sigma Aldrich) and 50 mg of Ile, Leu, Val (Sigma Aldrich) per liter of M9 minimal medium. At the OD of 0.6 – 0.8, 60 mg of L-Selenomethionine (Sigma Aldrich) was supplied to the medium while cells were induced by addition of 0.5 mM IPTG at 20°C for overnight. The SeMet ROC_ext_ purification was the same as wild-type ROC_ext_.

### Size-Exclusion Chromatography Coupled with Multi-angle Light Scattering (SEC-MALS)

To determine the absolute molecular weight of ROC_ext_ in solution, we used multiple angle light scattering. Our experimental setup includes an AKTA FPLC (GE Healthcare Biosciences, Piscataway, New Jersey) with a silica-based size-exclusion chromatography column (WTC-030S5, Wyatt Technology Corporation, Santa Barbara, California) as a liquid chromatography (LC) unit. Down from the LC is a refractive index detector (Optilab T-rEX, Wyatt Tech.) followed by a multiple light scattering detector (Dawn HeleosII, Wyatt Tech.) for determining protein concentration and particle size, respectively. Each sample injection consisted of ~1 mg of purified ROCext in buffer containing 30 mM HEPES (pH 7.4), 0.15 M NaCl, 10 mM MgCl_2_, 10 mM Glycine, 1 mM DTT, and 10% Glycerol. The flow rate was set at 0.4 mL/min and data were collected in a 1-second interval. Data processing and analysis were performed using the ASTRA software (Wyatt Tech.)

### Circular Dichroism Spectroscopy

CD spectra were collected on a Biologic Science Instruments MOS450 AF/CD spectrometer with a slit width of 1.0 mm and data acquisition of 1.0 s. The protein samples with concentrations ranging 0.46 – 0.86 mg/mL (based on absorbance at 280 nm) were dissolved in the buffer containing 10 mM Tris-HCl (pH 7.4), 150 mM NaCl, 5 mM MgCl_2_, 1 mM DTT, and 5% Glycerol.

### Fluorescence Polarization Nucleotide-binding Assay

To estimate the binding affinity of guanine nucleotides BODIPY-FL-GTPgammaS (100 nM) or BODIPY-FL-GDP (150 nM) (Molecular Probes) were titrated with ROC_ext_ (starting at 0.1 M) until saturation was reached (15 μM and 10 μM, respectively). Fluorescence polarization signals were read using an EnVison 2102 Multilabel Plate Reader (Perkin Elmer, Massachusetts) with excitation at 485 nm and emission at 535 nm. Experiments were performed at 25 °C in buffer containing 30 mM HEPES (pH 7.4), 150 mM NaCl, 10 mM MgCl_2_, 10 mM Glycine, 4 mM EDTA, 1 mM DTT, and 10% Glycerol. Data were analyzed using Prism 6 (GraphPad Software, CA, USA).

### GTPase Activity Assay

GTPase activity of ROC_ext_ was assessed by using the Enzcheck assay kit (Invitrogen) according to the manufacturer’s instructions. Briefly, ROCext (30 μM) was incubated with 2 mM GTP in buffer containing 30 mM HEPES (pH 7.4), 150 mM NaCl, 10 mM MgCl_2_, 10 mM Glycine, 1 mM DTT, and 10% Glycerol at 25 °C. Absorbance at 360 nm was recorded in every 3 minutes for 3 hours using a microplate reader. The amount of inorganic phosphate released from GTP hydrolysis at each time points was determined by extrapolation using a phosphate standard curve. In experiments comparing the activity of the S-S mutant with WT, DTT was omitted from the reaction for both the S-S mutant and WT. Data analysis and curve fitting were done with GraphPad Prism 7.

### Thermofluor Assay

Solutions of 12.5 μL of 10x Sypro Orange (prepared from 5,000x stock concentrate, Molecular Probes) in buffer containing 30 mM HEPES pH 7.4, 0.15 M NaCl, 10 mM MgCl_2_, 10 mM Glycine, 1 mM DTT, 4 mM GDP or Gpp(NH)p, 2 μM LMNG, and 10% Glycerol, and 12.5 μL of 25 μM ROC_ext_ or mutants were added to a 96-well thin-wall PCR plate. The plate was heated in the Real-Time PCR Detection System (Mastercycler realplex, Eppendorf) from 20 to 85 °C and fluorescence recorded in increments of 0.4 °C. The emission wavelength was set at 550 nm.

### Homology Modeling and Molecular Simulation

Homology models of the monomeric ROC domain in GDP-/GTP-bound and apo conformations were built based on the structures of Ras and *C. tepidium* Roco (PDB ID: 4Q21, 6Q21, 3DPU, and 6HLU, respectively) by using the program Modeller 9.19 (Andrej Sali, UCSF, San Francisco, CA). Molecular simulation of the S-S mutant was performed using CHARMM(30) and NAMD(27), and Molecular graphics display and presentation were made using PyMol (www.pymol.org).

### Cryo-EM Sample Preparation and Data Collection

Quantifoil UltrAuFoil R 1.2/1.3 gold grids (300 mesh) were glow-discharged for 1 min at 15 mA using a PELCO easiGLOW system. Purified full-length LRRK2 (2 mg ml^−1^, 7 μM) was applied to the grids (3 μl), blotted for 14 s with a blot force of 4 and plunge-frozen in liquid ethane using a Mark IV Vitrobot (Thermo Fisher Scientific).

Cryo-EM data were collected on a 200-kV Glacios transmission electron microscope (Thermo Fisher Scientific) equipped with a Falcon 4 direct electron detector. Automated data acquisition was performed using EPU software. Movies were recorded in electron-event representation (EER) mode at a nominal magnification of 150,000×, corresponding to a calibrated pixel size of 0.93 Å. A total of 2,945 movies were collected over a defocus range of −0.8 to −2.5 μm with a total electron dose of 50 e^−^ Å^−2^.

### Cryo-EM Data Processing

Movies were processed in CryoSPARC v4.7.1 where the raw movies underwent Patch Motion Correction followed by Patch Contrast Transfer Function (CTF) estimation. Micrographs were analyzed within the Curate Exposures job using motion trajectory output and CTF fit values, and poor micrographs were discarded (2,324 accepted micrographs out of 2,945). Particles were initially picked using Blob Picker and extracted with a binning factor of 2, for reference-free 2D classification. Three groups of classes were observed, and the best classes for each group were selected (11,000, 9,000, and 12,000 particles per group) for Topaz training. Particles picked by iterative Topaz training were extracted with a binning factor of 2 and several rounds of 2D classification were performed to eliminate obvious noise. Well-defined classes for each observed group were selected (53,000 particles) and used for *ab initio* reconstruction, resulting in three distinct volumes. Heterogeneous Refinement with the three *ab initio* volumes and a junk volume was performed with all topaz selected particles to further classify the particles belonging to each conformation, resulting in 18,032 particles in autoinhibited conformation, 18,282 particles in the intermediate conformation, and 30,783 particles in the activated conformation. The particles for each class were re-extracted without binning and refined using Non-Uniform Refinement. All reported resolutions were calculated based on the gold-standard Fourier Shell Correlation (GSFSC) cut off at 0.143.

### Crystallographic data collection and structure determination

A 1.6 Å data set from crystals of ROC_ext_ was collected at beamline 23-ID-D of the Advanced Photon Source at Argonne National Laboratory. The X-ray wavelength was set at 1.03 Å. We cryo-cooled ROC_ext_ crystals in liquid nitrogen after transferring them to a cryo-protectant containing 10% glycerol, 100 mM KSCN, 25% PEG MME, and 0.1M BisTris, pH 6.5. Diffraction data were collected at 100 K on a Pilatus3 6M detector (DECTRIS, Switzerland) and processed with the program HKL2000(31). The crystals belonged to the space group *P*2_1_ with two molecules per asymmetric unit. The homology model was used as a molecular-replacement search model in combination with single anomalous dispersion (SAD) and yielded a solution with the program Phenix(21). A 2fo-2fc map following rigid body refinement showed reasonable density for ~80% of the model. Data of Se-Met ROC_ext_ crystals to 3.0 Å resolution were collected on the same beamline. The X-ray wavelength was set at 0.98 Å. Cycles of rebuilding and refinement were carried out using the programs Coot (32, 33), Phenix (21), and Refmac (34). The current *R*_cryst_ and *R*_free_ are 13.5% and 15.8%, respectively (r.m.s. deviations from ideal bond lengths and angles are 0.023 Å and 2.6°, respectively).

## Supplementary Material

1

## Abbreviations:

LRRK2: Leucine-Rich Repeat Kinase 2
ROC: Ras of complex proteins
COR: C-terminal of ROC
PD: Parkinson’s disease
