# Supplementary material for: Structural and biochemical basis of ROC-dependent activation of LRRK2
Source: Res Sq. 2026 Feb 2:rs.3.rs-8735353. Preprint. [Version 1] doi: 10.21203/rs.3.rs-8735353/v1 (PMC12889809; doi:10.21203/rs.3.rs-8735353/v1)

Table 1 Data collection and refinement

**Data collection**			
Data set	ROC_{KA}	ROC_{WT}	ROC_{WT}-SeM
Space group	$P2_1$	$P2_1$	$P2_1$
Unit cells	44.63 101.88 44.61<br>90.00 100.95 90.00	44.65 103.69 44.59<br>90.00 101.20 90.00	44.58 102.95 44.61<br>90.00 101.31 90.00
Wavelength (Å)	1.0331	1.0331	0.9794
Resolution (Å)[1]	40.26-1.59<br>(1.65-1.59)	43.8-1.95<br>(2.02-1.95)	51.47-3.00<br>(3.19-3.00)
Completeness (%)	97.37 (78.95)	98.64 (98.46)	98.1 (97.4)
R_{merge}[2]	0.084 (0.577)	0.072 (0.457)	0.064 (0.122)
R_{meas}[3]	0.087 (0.623)	0.078 (0.516)	0.090 (0.172)
R_{pim}[4]	0.023 (0.223)	0.031 (0.233)	0.064 (0.122)
$CC_{1/2}$	(0.804)	(0.874)	(0.937)
I/σ(I)	41.9 (1.7)	24.0 (2.3)	10.8 (6.2)
**Refinement**			
Unique reflection	50672 (4069)	28703 (2821)	
Protein atoms	2965	2987	
Solvent atoms	227	249	
Ligands	60	60	
R-factor (R_{free}) (%)[5]	15.40 (16.72)	16.17 (20.43)	
Average B-factor ($Å^2$)	40.61	50.89	
R.M.S. deviations			
Bonds (Å)	0.013	0.013	
Angles (°)	1.73	1.75	
**Ramachandran plot**			
most favored regions (%)	75.14	95.66	
Additionally allowed regions (%)	3.78	3.52	
Outlier regions (%)	1.59	0.81	

[1]Values for the highest resolution shell are indicated in parentheses.

[2] $R_{merge} = \Sigma_h\Sigma_i |I_{hi}-\langle I_h\rangle|/\Sigma_h\Sigma_i I_h$, where I_{hi} is the intensity of the ith observation of reflection h, and $\langle I_h\rangle$ is the average intensity of redundant measurements of the h reflections.

[3] $R_{meas} = \Sigma_h \sqrt{(n/(n-1)}\Sigma_i |I_{hi}-\langle I_h\rangle|/\Sigma_h\Sigma_i I_h$, where I_{hi} is the intensity of the ith observation of reflection h, and $\langle I_h\rangle$ is the average intensity of redundant measurements of the h reflections.

[4] $R_{pim} = \Sigma_h \sqrt{(1/(n-1)}\Sigma_i |I_{hi}-\langle I_h\rangle|/\Sigma_h\Sigma_i I_h$, where I_{hi} is the intensity of the ith observation of reflection h, and $\langle I_h\rangle$ is the average intensity of redundant measurements of the h reflections.

[5]R-factor = $\Sigma||F_o|-|F_c||/\Sigma|F_o|$, where F_o and F_c are the observed and calculated structure-factor amplitudes. R_{free} is monitored with 5% of reflections excluded from refinement.

Figure S1. Data processing workflow using CryoSPARC.

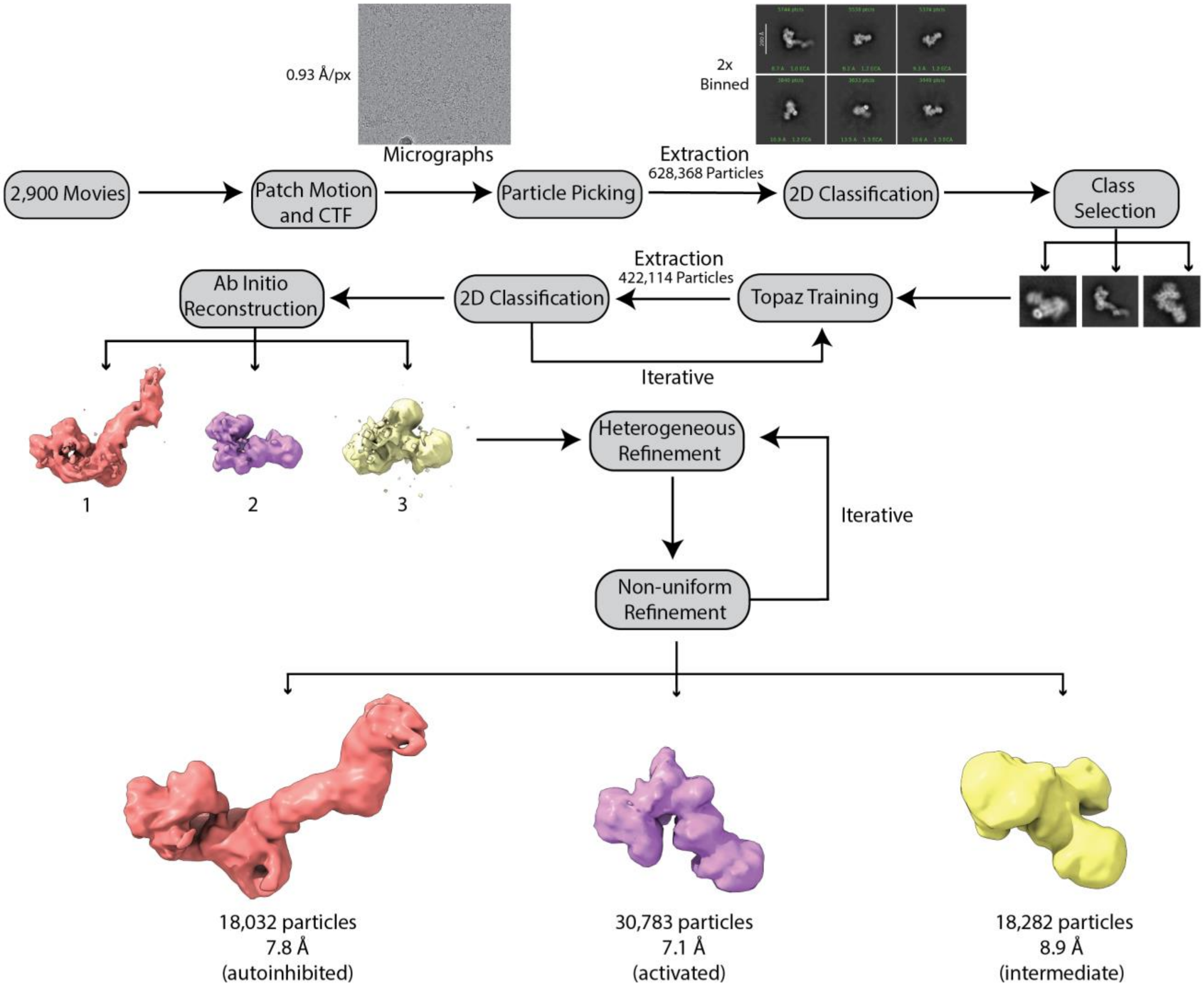

Figure S2. Gold standard FSC curve for full-length LRRK2.

Activated

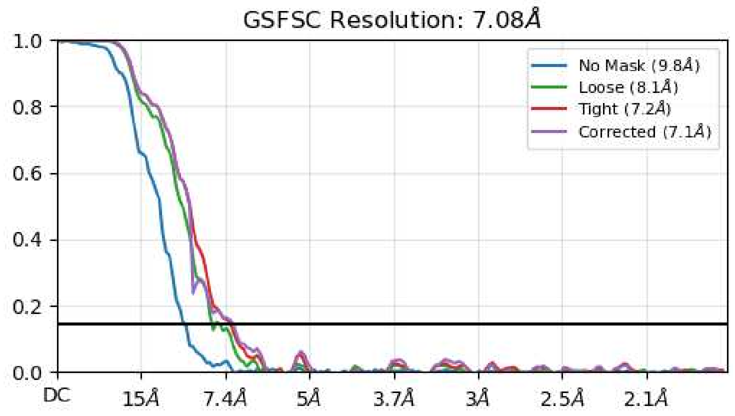

Autoinhibited

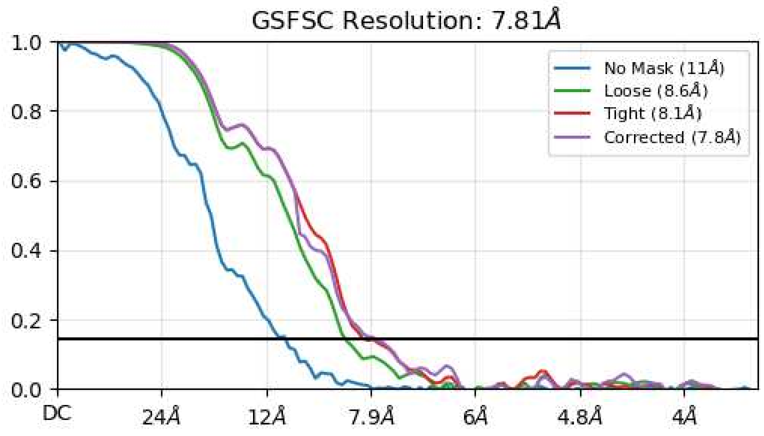

Intermediate

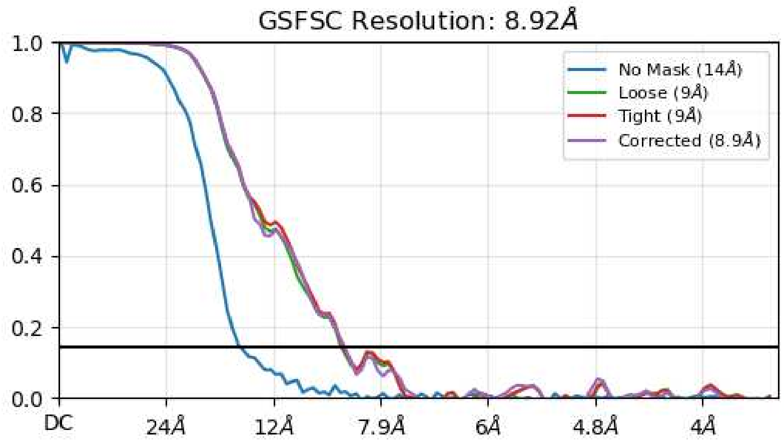

Figure S3. Arrangement of domains in monomeric full-length LRRK2. Surface presentation of the autoinhibited, intermediate, and activated models of LRRK2 with the EM map mesh. Showing the LRR domain (yellow) of the intermediate conformation is partially disengaged from the kinase domain (purple).

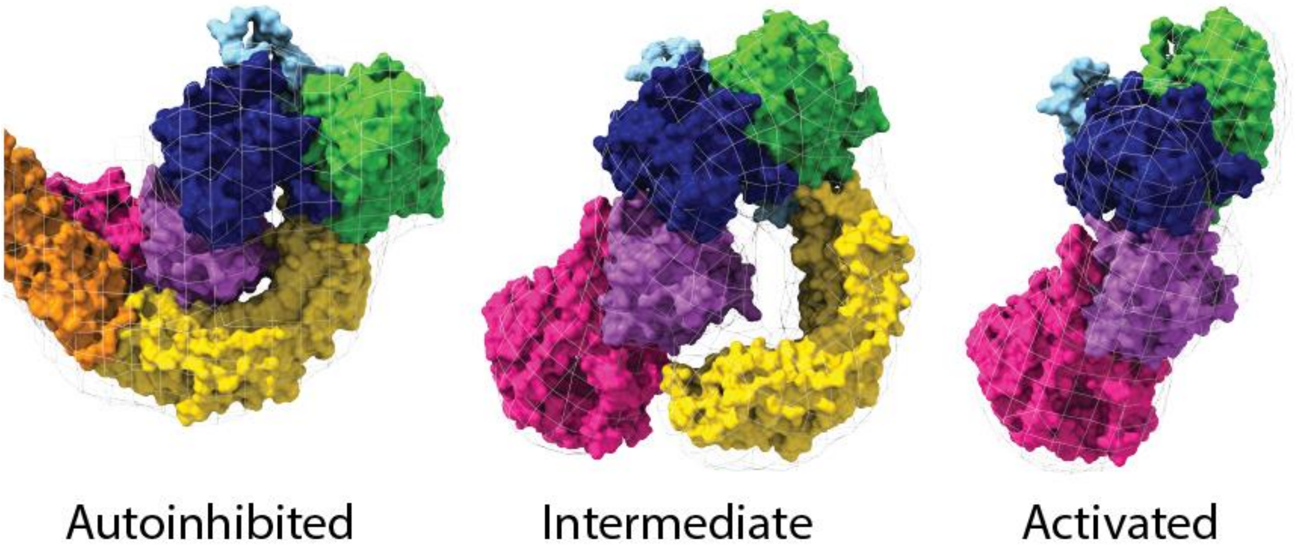

Figure S4. Arrangement of the COR domain in monomeric full-length LRRK2. Surface presentation of the autoinhibited, intermediate, and activated models of LRRK2. Showing the COR domain of the autoinhibited (dark blue), intermediate (medium blue), and activated (light blue) conformations relative to the superposed kinase domain (purple).

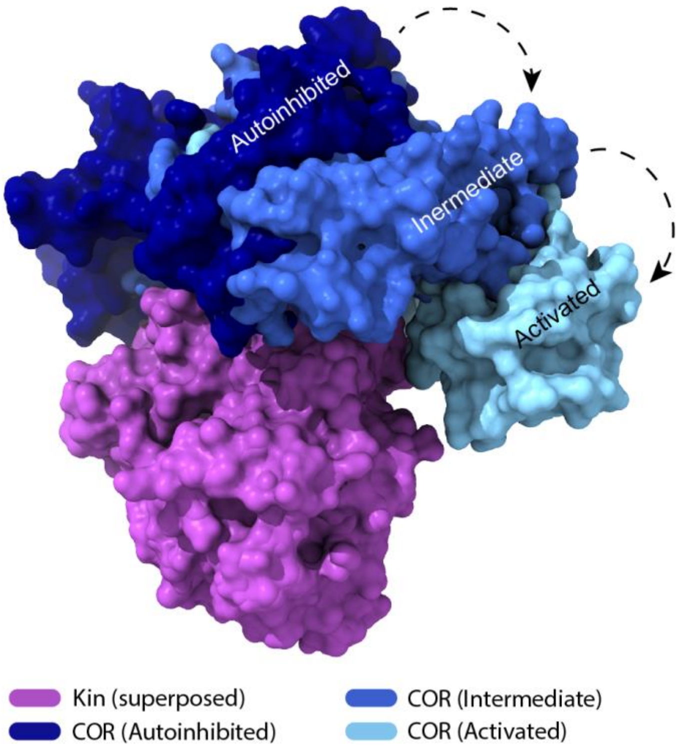

Figure S5. Structure and activity of WT vs. KA surface engineered ROC$_{ext}$. a) Superposition of WT (gold) and a surface-engineered (K1460A, K1463A) construct of ROCext (green), showing that the two structures are practically the same. **b**) GTPase activity of the surfaced engineered mutant (KA, red), WT (blue), and a PD-associated mutant R1441H (green); showing that the surface engineering did not significantly affect its GTPase activity compared to the WT.

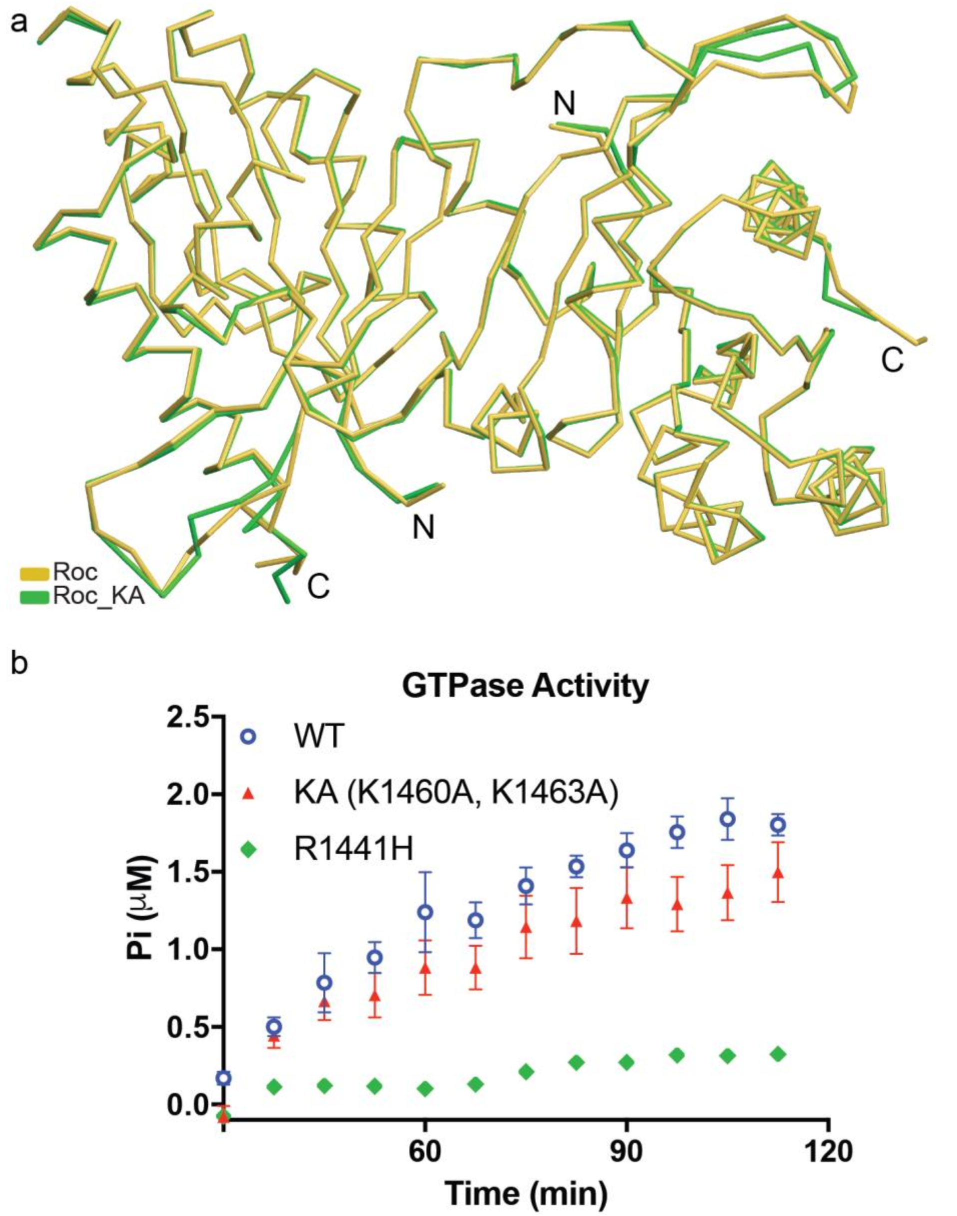

Figure S6. Structure of Rocext. **a**) Crystal structure of ROC dimer (1.6 Å) shown in semi-transparent surface and ribbon presentations. Chain A is colored orange and chain B colored teal. The dotted lines outline the areas presented in **b**) and **c**). **b**) Enlarged view of the area highlighted in top panel of **a**) showing extensive hydrogen bonding at the dimer interface. **c**) Enlarged view of the area highlighted in bottom panel of **a**) showing a hydrophobic patch at the dimer interface, which is capped at both ends by residue R1441.

.

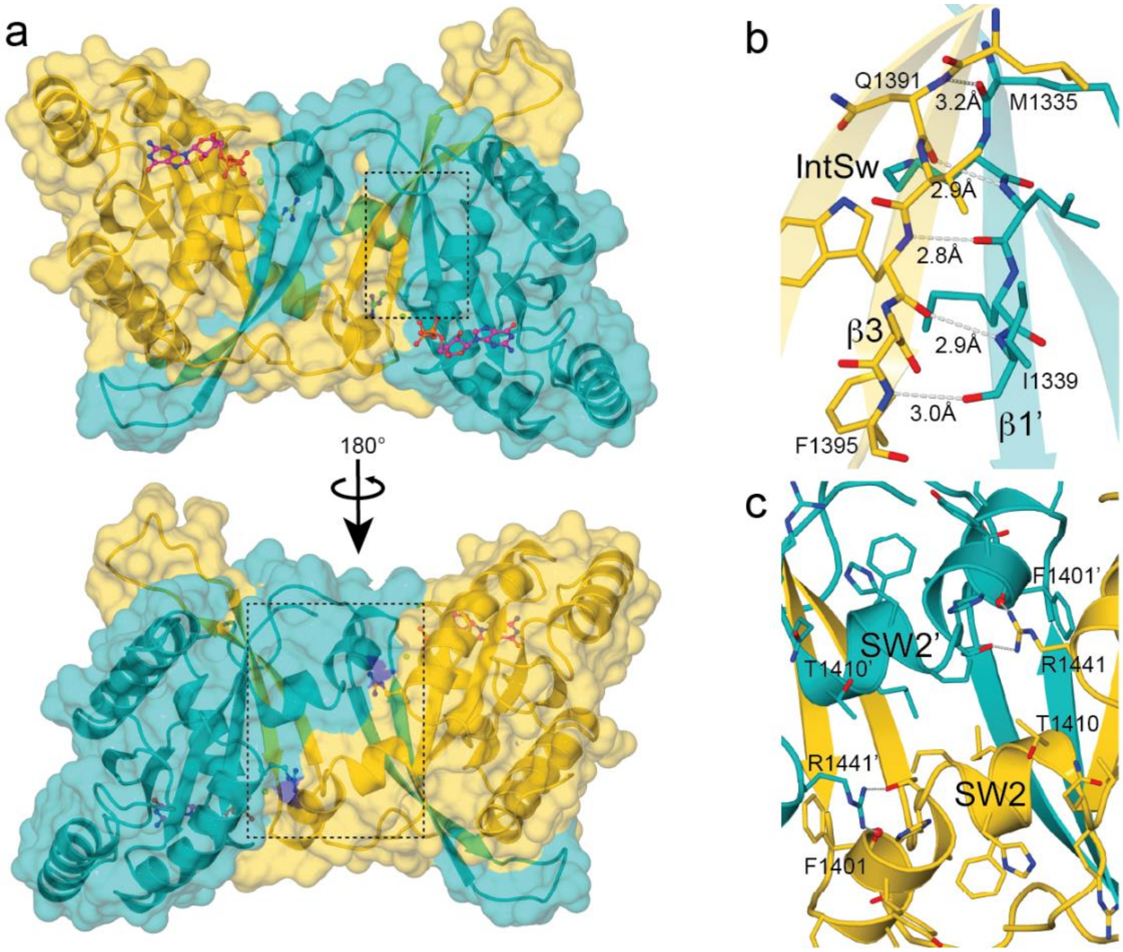

Figure S7. Molecular simulation of disulfide-stabilized ROC$_{ext}$. **a**) Ribbon presentation of a molecular dynamic simulation model of ROC dimer consisting of an engineered disulfide bond between residues 1398 and 1431 (S-S). **b**) Crystal structure of ROC$_{ext}$ (light grey) superimposed with the calculated S-S dimer (gold and teal) (RMSD 0.93 A^2), showing no significant structural changes upon disulfide formation. The engineered disulfide bonds are highlighted with dotted lines.

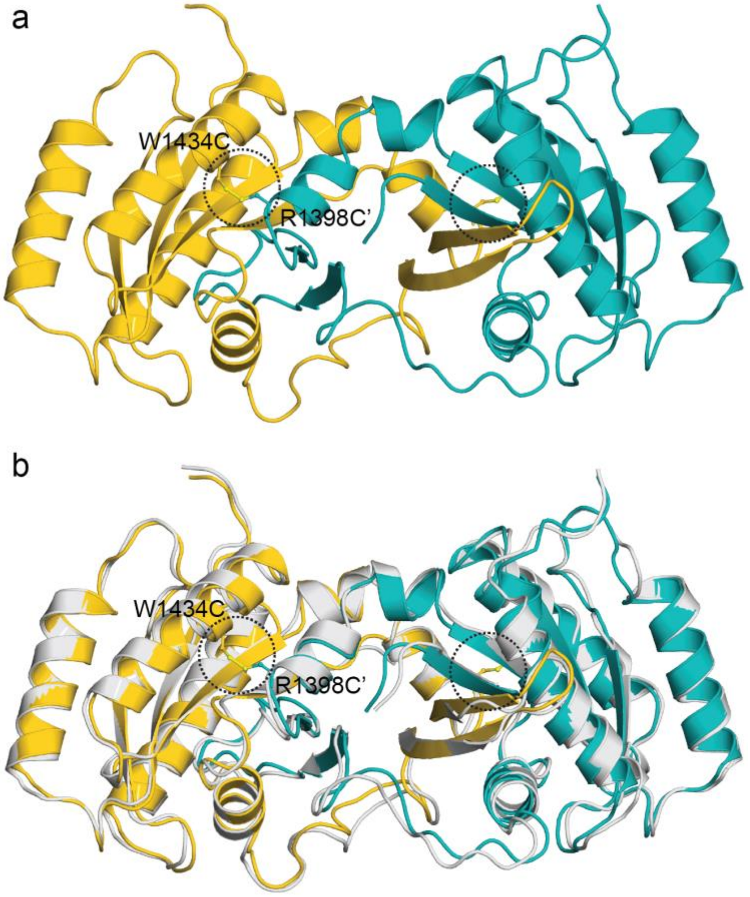

Figure S8. Molecular simulation of disulfide-stabilized ROC$_{ext}$. a) Ribbon presentation of the dimer interface of ROCext showing the interactions of residue R1441. **b**) Ball and stick model of ROC$_{ext}$ showing residue R1441 interactions at the dimer interface. Electron density countered at 1 σ. **c**) SEC-MALS of the R1441K mutant form of ROC (blue line) compared to the WT dimer (black line) and WT monomer (black dotted line), showing that substituting arginine for lysine at position 1441 completely abolished dimerization. **d**) GTPase activity of R1441K (purple and blue) compared to WT (black), showing that the R1441K mutation impaired GTPase activity.

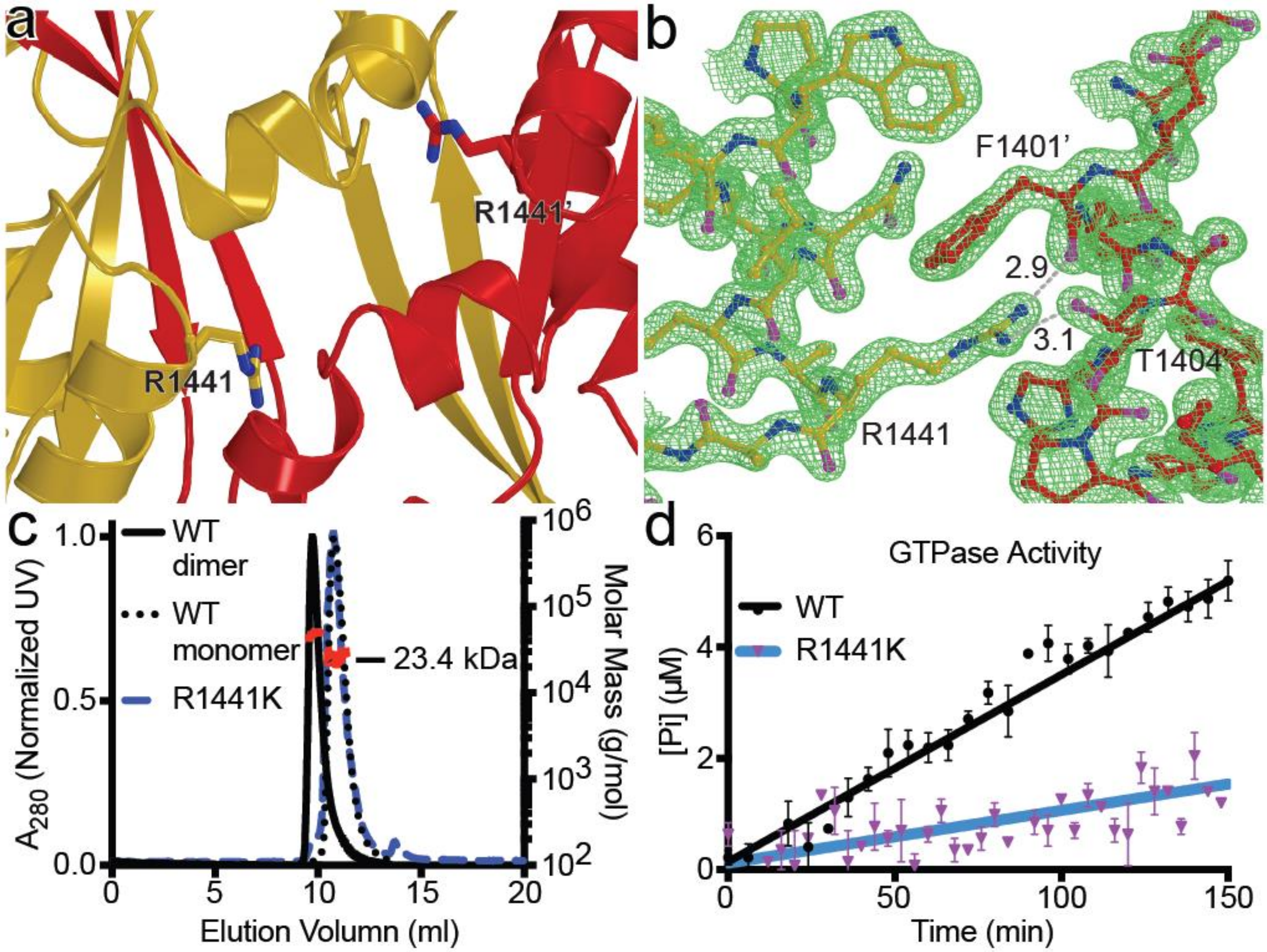

Figure S9. Disulfide-stabilized dimer. Size-exclusion chromatography coupled with multi-angle light scattering, showing that the elution profile and calculated mass of the disulfide-stabilized dimer (dashed line) was comparable to wild-type dimer (solid black line).

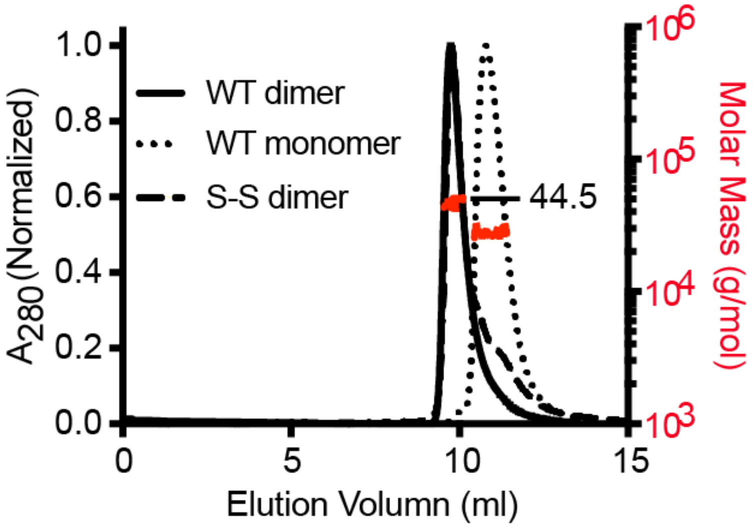

Figure S10. SDS-PAGE of disulfide-stabilized dimers and monomers. **a**) Non-reducing SDS-PAGE showing the S-S monomer (S1) migrating at about 22 kDa and the S-S dimer (S2) at about 44 kDa. **b**) Reducing SDS-PAGE showing that both monomers (S1) and dimers (S2) migrating at about 22 kDa.

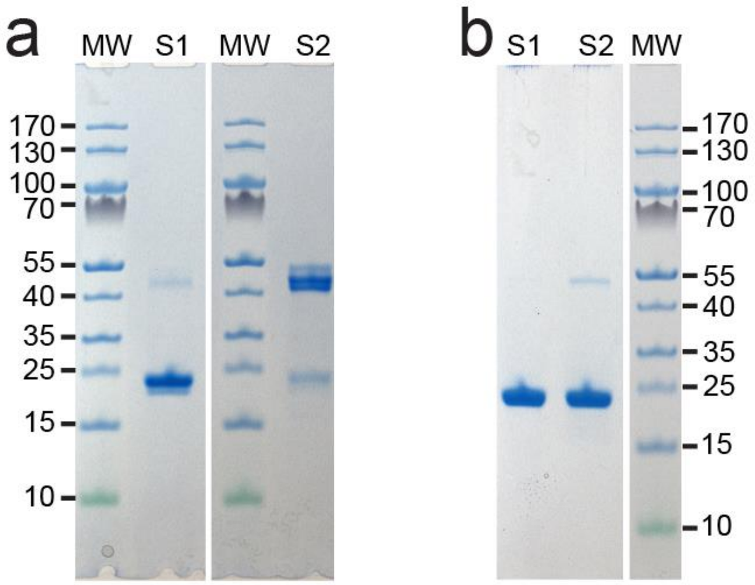

Figure S11. Crystals of WT and the surface-engineered KA construct. Optimized crystals of WT ROC_{ext} (left panel) and the surface-engineered K1460A-K1463A (right panel), showing improvement of crystals from clusters of thin plates (left panel) to a single crystal with increased thickness along the b-axis (right panel).

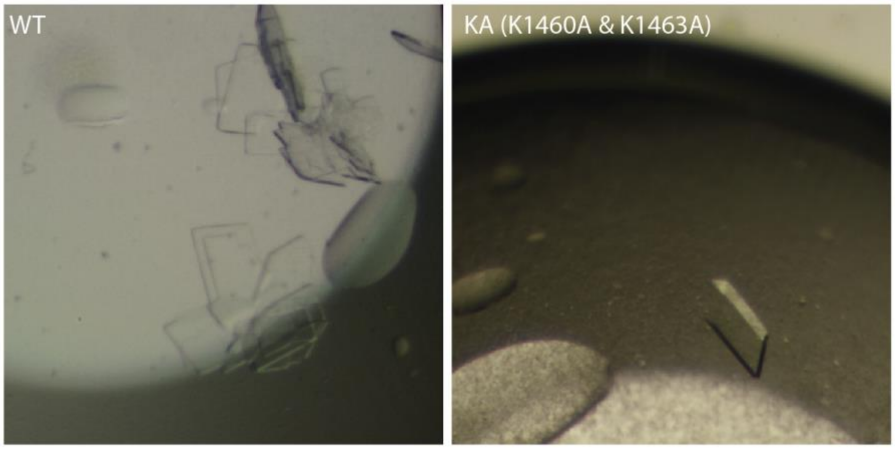

Supplement: 1 [file NIHPPRS8735353V1-supplement-1.pdf]
